# N-acetylcysteine attenuates sodium arsenite-induced oxidative stress and apoptosis in embryonic fibroblast cells

**DOI:** 10.1093/toxres/tfae128

**Published:** 2024-08-13

**Authors:** Tunahan Tasci, Banu Orta-Yilmaz, Yasemin Aydin, Mahmut Caliskan

**Affiliations:** Department of Biology, Institute of Graduate Studies in Sciences, Istanbul University, Istanbul 34126, Turkey; Department of Medical Services and Techniques, Vocational School of Health Services, Istanbul Bilgi University, Istanbul 34387, Turkey; Department of Biology, Faculty of Sciences, Istanbul University, Istanbul 34126, Turkey; Department of Biology, Faculty of Sciences, Istanbul University, Istanbul 34126, Turkey; Department of Biology, Faculty of Sciences, Istanbul University, Istanbul 34126, Turkey

**Keywords:** N-acetylcysteine, sodium arsenite, oxidative stress, apoptosis, embryonic fibroblast cells

## Abstract

In recent years, the increase in environmental pollutants has been one of the most important factors threatening human and environmental health. Arsenic, a naturally occurring element found in soil, water, and air, easily enters the human body and leads to many metabolic disorders. In this study, we focused on the possible protective effects of N-acetylcysteine (NAC) against sodium arsenite (As)-induced toxic effects on embryonic fibroblast cells. The effects of As and NAC treatment on cells were evaluated, including cytotoxicity, oxidative stress, and apoptosis. Embryonic fibroblast cells were exposed to As (ranging from 0.01 μM to 10 μM) and NAC (at a concentration of 2 mM) for 24 h. The assessment of cytotoxicity markers, such as cell viability and lactate dehydrogenase (LDH), showed that As significantly reduced cell viability and increased LDH levels. Furthermore, we observed that As increased the amount of reactive oxygen species (ROS) in the cell, decreased the activity of antioxidant enzymes, and triggered apoptosis in cells. Additionally, our research revealed that the administration of NAC mitigates the detrimental effects of As. The results showed that As exerted hazardous effects on embryonic fibroblast cells through the induction of oxidative stress and apoptosis. In this context, our study provides evidence that NAC may have a protective effect against the toxicity of As in embryonic fibroblast cells.

## Introduction

Arsenic, one of the most biohazardous metalloids, is a toxic element that largely disperses into the environment and accumulates in the earth’s crust.^1–3^ Arsenic is widely used in various sectors, including industry, medicine, and agriculture. However, the exposure to arsenic has emerged as a worldwide health concern for all living organisms.^1^ Arsenic and its derivatives are found in drinking water, food, soil, and air particles.^4^ Recent research has demonstrated that the consumption of drinking water contaminated with arsenic has a significant impact on the overall health of a large population.^2^^,^^5^ Although the World Health Organization (WHO) establishes the acceptable level of arsenic in water as 10 μg/L, this level is between 0.5 and 5,000 μg/L in America, India, Nepal, Canada, Argentina, Turkey, and China.^3^

Arsenic, a classified elemental group I carcinogen with toxic effects on most organ systems, has emerged as a major environmental issue impacting millions of people today.^5^^,^^6^ Arsenic causes toxicity by reacting with thiols in cells, leading to the formation of reactive oxygen species (ROS), cellular damage, and disruption of intracellular enzyme systems. Many studies in the literature show that different types of reactive oxygen species (ROS) arise due to arsenic metabolism.^4^^,^^6^ While arsenic-induced oxidative stress causes damages to organs such as the pancreas, liver, kidney, testis and lung, it is also associated with various diseases such as cancer, atherosclerosis, and cardiovascular system disorders.^6^^,^^7^ There are many studies investigating the effects of arsenic on fibroblast cells.^8–13^ In a study examining the effects of arsenic on human fibroblast cells, it was revealed that arsenic disrupts glutathione (GSH) release and causes chromosome damage.^10^ Another study found that arsenic triggered cell death by increasing the amount of ROS in human pulmonary fibroblasts.^11^ Arsenic induced apoptosis in cardiac fibroblast cells by altering the expression levels of apoptosis-related genes and proteins, according to a previous study.^12^ An in vitro study demonstrates that arsenic triggers apoptosis in human pulmonary fibroblasts by modifying the expression of the *transformation-related protein 53* (*Trp53*) gene and the *caspase-3* (*Casp3*) gene.^13^

NAC is recognized by the WHO as an essential drug in the primary health care system. NAC is a safe antidote in various doses and is used for glutathione deficiency in a wide variety of metabolic disorders, such as lung diseases, neurotoxicity, hepatotoxicity, and immunotoxicity.^14^ As an antioxidant, NAC neutralizes free radicals without causing damage to cells and it can act as an electron pair donor within the cell.^15^ NAC is a direct precursor of glutathione synthesis and is a dietary supplement that regenerates intracellular glutathione.^16^ Research has shown that NAC can reduce pro-inflammatory cytokines within the cell, induce neurogenesis, improve mitochondrial function, and regulate apoptosis.^17^ It is also known to have some clinical benefits as a chelating agent in the treatment of acute poisoning by heavy metals, including arsenic, both as a substance that can protect the liver and kidney from damage and as an intervention tool to increase the elimination of metals.^18–20^

Fibroblasts are the most common cells in connective tissue and are responsible for the synthesis of extracellular matrix components.^21^ Fibroblasts not only possess supporting and wound-healing characteristics, but also play important functions in regulating organ development, inflammation, and fibrosis. These cells, found in every tissue, share properties with mesenchymal bone marrow-derived stem cells and are particularly sensitive due to their embryonic origin.^22^ Because of these properties, fibroblasts are an interesting in vitro model for cell biology studies. Researchers have extensively studied the effects of arsenic on various cell types, but it's crucial to explore how arsenic exposure affects basic cell function in embryonic fibroblast cells. In this study, we investigated the possible protective effects of NAC against arsenic toxicity on embryonic fibroblast cells. In this context, cell cytotoxicity, ROS production, and antioxidant enzymes activities were determined in embryonic fibroblast cells treated with arsenic and/or NAC. Furthermore, to illustrate the apoptotic effects, apoptotic cells were labeled using the double fluorescence labeling technique, and the levels of gene expression for *B-cell lymphoma 2 (Bcl2), Bcl-2 associated X-protein (Bax), Trp53*, and *Casp3* were quantified. Consequently, it was investigated for the first time whether NAC had a protective effect on cellular damage caused by As in embryonic fibroblast cells.

## Materials and methods

### Cell culture and exposure

3T3 embryonic fibroblast cells were obtained from the Global Bioresource Center, American Type Culture Collection (ATCC, Manassas/Virginia, USA). Cells were maintained in DMEM culture medium with 10% calf serum, 4.5 g/L glucose, L-glutamine, sodium pyruvate, and Penicillin–Streptomycin-Amphoterine (PSA) in a humid environment containing 5% CO_2_ and 95% air at 37 °C. Sodium arsenite (As) was purchased from Molychem (Cat. No. 25480) (Maharashtra, India). As (0.01, 0.1, 1, and 10 μM) prepared in cell culture medium and was applied in the presence and absence of NAC to cells for 24 h. Concentrations of As (0.01–10 μM) were determined using data from Gomez-Caminero et al.^23^ and Petrusevski et al.^24^ These studies evaluated the levels of As that humans could be exposed to in their blood serum after being exposed to the environment. In this investigation, we selected a 2 mM concentration of NAC as the optimal therapeutic concentration. This concentration has been previously utilized in in vitro experiments.^25^^,^^26^

### Cytotoxicity

To determine cytotoxicity, 5 × 10^3^ embryonic 3T3 fibroblast cells per well were seeded in 96-well plates for the cell viability assay (MTT). The 3T3 cells were analyzed using a 3-(4,5-dimethylthiazol-2-yl)-2,5-diphenyltetrazolium bromide (MTT) kit (Roche Molecular Biochemicals, Mannheim, Germany). A volume of 10 μL of MTT I solution was added to each well of a 96-well culture plate at the end of the treatment period. Subsequently, the culture plates were incubated in a CO_2_ incubator at 37 °C for 4 h to convert the MTT dye into water-insoluble formazan crystals. To dissolve the formazan crystals formed by living cells, 100 μL of MTT II solution (SDS) was added to each well and left overnight in a CO_2_ incubator. At the end of this period, the optical densities of the solution formed were read using an ELISA reader at a wavelength of 540 nm. Cell viability in the control group was assumed to be 100%, and the viability rates of the experimental groups were expressed as a relative percentage.

The lactate dehydrogenase test (LDH) was applied to detect cellular proliferation of embryonic 3T3 fibroblast cells seeded with 1 × 10^4^ cells/well in 96-well plates. LDH is an intracellular enzyme that passes to the external environment (culture media) in cases of damage to the cell membrane. After the application of As and NAC to cells for 24 h, 100 μL of medium was placed in 96-well plates, and 100 μL of LDH assay solution was added to determine LDH in the medium colorimetrically. After the reagent mixture was prepared, it was incubated at room temperature for 30 min in the dark, and the measurement was performed to measure absorbance at 492 nm using an ELISA reader. The cytotoxicity of the experimental groups was shown as a percentage, assuming the cytotoxicity of the control cells was 100%.

### Biochemical analysis

For biochemical tests, 5 × 10^5^ cells were seeded in six-well plates, and at the end of exposure, cells were treated with trypsin–EDTA and transferred to Tris–HCl buffer (pH 7.2). The cell lysate was obtained by sonication and centrifuged for 15 min at 14,000 g at 4 °C. The supernatant obtained was used for hydroxyl radical (OH•), malondialdehyde (MDA), superoxide dismutase enzyme (SOD), catalase (CAT), glutathione peroxidase (GPx), glutathione-S-transferase (GST), and Lowry assays.

### Measurement of oxidative stress markers

The determination of the OH• was achieved according to the method of Puntarulo and Cederbaum.^27^ This method is based on the generated OH• in the presence of NADPH and DMSO. The generated formaldehyde was allowed to react with trichloroacetic acid and the absorbance was measured at 570 nm.

The level of lipid peroxidation was measured based on the MDA content using the method of Devasagayam and Tarachand.^28^ The experimental principle is based on measuring the compound formed using MDA as a substrate with thiobarbituric acid at a wavelength of 532 nm.

### Measurement of antioxidant enzymes

The method of Marklund and Marklund^29^ was used to measure the activity of SOD. The principle of the experiment relies on the auto-oxidation of pyrogallol, which is inhibited by the SOD enzyme. After the assay mix was prepared, the sample and blank tubes were read at 420 nm for 3 min, and the results were reported as units/mg of protein. The method of Sinha^30^ was used to measure CAT activity based on the principle that the precipitate formed by the dichromate/acetic acid indicator is H_2_O_2_ at 570 nm in the ELISA reader (Thermo Scientific, Waltham, MA, USA). Enzyme activity is measured in μM of H_2_O_2_ consumed per protein. The method described by Hafeman et al.^31^ was used to demonstrate GPx enzyme activity based on the absorbance detected at 412 nm wavelength of a compound formed following exposure to 5,5′-dithio-bis (2-nitrobenzoic acid) glutathione, which is a substrate of the GPx enzyme. The method of Habig et al.^32^ was used to demonstrate GST. The GST enzyme forms a glutathione-DNB conjugate in the presence of glutathione and 1-chloro-2,4-dinitrobenzene. Enzyme activity is indicated as μmol CDNB-glutathione mg/protein formed per min.

### Analysis of apoptosis

The percentage of viable, apoptotic, and dead cells was assessed by double-fluorescent staining with fluorescent dyes capable of binding to DNA. The fluorescent dye Hoechst 33,342 stains the condensed chromatin in apoptotic cells blue, and the red fluorescent dye propidium iodide, which binds only to the DNA of cells with impaired membrane integrity, was used. 3T3 cells (2 × 10^4^ cells per well) were seeded in 24-well plates, and at the end of the exposure time, the cells were washed with phosphate buffered saline (PBS). Immediately afterwards, cells were incubated with a 1:1 (1 mg/mL) solution of propidium iodide/Hoechst 33,342 in PBS at 37 °C for 15 min. Cells were washed several times with PBS after incubation, examined with an Olympus IX71 fluorescence microscope and ultraviolet filter (Tokyo, Japan), and photographed serially with an Olympus DP72 video camera (Tokyo, Japan) at equal time intervals. The ratio of live, apoptotic, and dead cells was calculated by counting a total of 1,000 cells for each experimental group in the serial photographs.

To determine the expression of apoptotic and pro-apoptotic genes, 3T3 cells were seeded in six-well plates with 1 × 10^6^ cells per well. After treatments, total RNA was isolated with a Total RNA isolation kit (GENAXXON, Ulm, Germany) according to the manufacturer’s instructions. The ND-2000c Nanodrop (Thermo Scientific, Darmstadt, Germany) was used to measure the A260/A280 ratio, which was detected between 1.8 and 2.0. First-strand complementary DNA was synthesized according to the manufacturer's instructions with the Advanced cDNA Synthesis Kit (WISENT, Quebec, Canada) and random primers. The primers used for real-time PCR are listed in Table 1. Reverse transcription was performed at 25 °C for 10 min as the pre-incubation step, followed by a 30-min incubation at 42 °C, and the reaction was terminated by keeping the samples at 85 °C for 10 min. Real-time PCR reactions were performed in triplicate for each sample using the Light Cycler 480 system (Roche) device. The Lightcycler SYBR Green kit (Roche Applied Science, Mannheim, Germany) was used according to the manufacturer’s instructions for measuring gene expression. The housekeeping gene β-actin was used as an internal control. Thermal cycle steps were carried out at 95 °C for 5 min, followed by 45 cycles at 95 °C for 10 s, 30 s for primer binding, and 25 s at 72 °C for strand elongation. The Livak and Schmittgen^33^ 2^−ΔΔCt^ method was used to analyze the data, and fold changes between As and NAC-treated groups were shown relative to the control groups.

**Table 1:** Primer sequences for RT-PCR.

**Primer**	**Forward**	**Reverse**
*Bcl2*	5′-ATGGGGTGAACTGGGGGATTG-3′	5′-TTCCGAATTTGTTTGGGGCAGGTC-3′
*Bax*	5′-GGGTGGTTGCCCTTTTCTACT-3′	5′-CCCGGAGGAAGTCCAGTGTC-3′
*Casp3*	5′-CTTGGTAGATCGGCCATCTGAAAC-3	5′-GGTCCCGTACAGGTGTGCTTCGAC-3′
*Trp53*	5′-GGAGTATTTGGACGACCG-3′	5′-TCAGTCTGAGTCAGGCCC-3′
β*-Actin*	5’-CGTTGACATCCGTAAAGAC-3′	5′-TGGAAGGTGGACAGTGAG-3′

### Statistical analysis

All data processing and statistical analysis were performed using GraphPad 10 software (GraphPad Software, San Diego, CA). Tukey’s multiple comparison test and one-way analysis of variance were used on data from three different experiments in triplicate. Results were expressed as the mean ± standard error of the mean. The normality of the data distribution was evaluated using the Shapiro–Wilk test. *P* < 0.05, *P* < 0.01, and *P* < 0.001 values were considered statistically significant.

## Results

### NAC ameliorated cytotoxicity induced by As

**Fig. 1:**
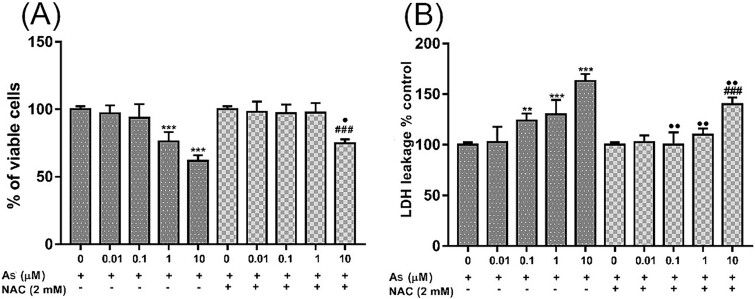
Effects of As and NAC concentrations on the viability (A) and LDH activity (B) of embryonic fibroblast cells in 24 h. Each bar represents the results of the four replicates mean ± SEM of three independent experiments. Significance was shown as ^*^^*^P < 0.01, ^*^^*^^*^P < 0.001 compared to the control, ^###^*P* < 0.001 compared to NAC alone, ^•^*P* < 0.05, ^••^*P* < 0.01 compared to As alone groups. As; sodium arsenite, NAC; N-acetylcysteine.

Cell viability was determined by the MTT assay after the exposure of As and NAC to embryonic 3T3 fibroblast cells. Cell viability was found to be significantly decreased in the 1 μm and 10 μm As groups compared to the control (^*^^*^^*^P < 0.001) (Fig. 1A). Furthermore, an ameliorative effect of NAC was observed when NAC was added to the 10 μm As group (^•^*P* < 0.05).

The damage to the cell membrane was determined by measuring the LDH enzyme in the medium. When we evaluated the groups exposed to As in terms of LDH activity, a significant increase was found at the 0.1 (^*^^*^P < 0.01), 1, and 10 μm concentrations (^*^^*^^*^P < 0.001) (Fig. 1B). In addition, it was discovered that NAC effectively inhibited the release of LDH at a concentration of 10 μM As (^###^*P* < 0.001).

### NAC suppressed the oxidative damage induced by As

**Fig. 2:**
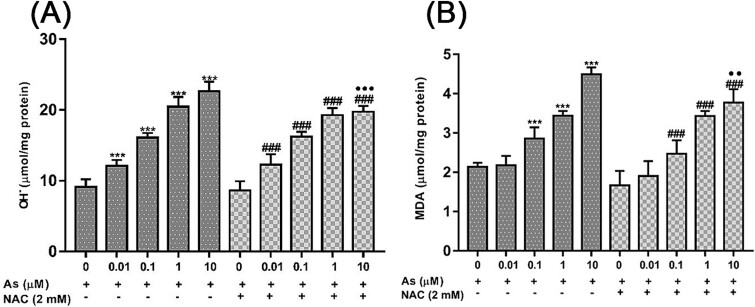
The effects of As and NAC on hydroxyl radical levels (A) and lipid peroxidation (B) of embryonic fibroblast cells at 24 h. Each bar represents the four replicates mean ± SEM of three independent experiments. Significance was shown as ^*^^*^P < 0.01, ^*^^*^^*^P < 0.001 compared to the control, ^###^*P* < 0.001 compared to NAC alone, ^••^*P* < 0.01, ^•••^*P* < 0.001 compared to As alone groups. As; sodium arsenite, NAC; N-acetylcysteine.

The levels of two oxidative stress markers were evaluated following the administration of As and NAC on embryonic fibroblast cells in Fig. 2. When OH• and MDA were evaluated compared to the control group, it was determined that they increased significantly at all As concentrations used (except for the 0.01 μM concentration for MDA) (^*^^*^^*^P < 0.001). When the ameliorative effects of NAC were evaluated, it was observed that the findings of both OH• radical (^•••^*P* < 0.001) and MDA (^••^*P* < 0.01) levels decreased significantly at 10 μM As concentrations.

### NAC improved antioxidant enzymes induced by As

**Fig. 3:**
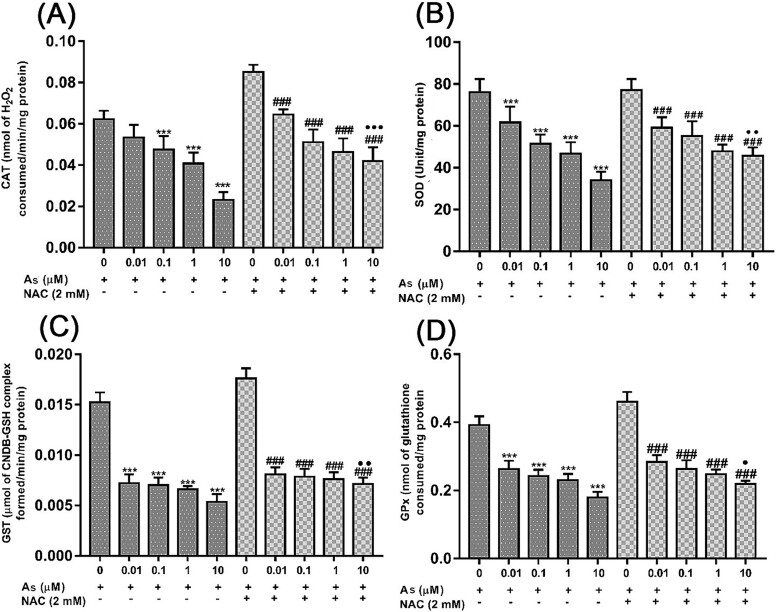
Effects of As and NAC on CAT (A), SOD (B), GST (C) and GPx (D) of embryonic fibroblast cells in 24 h. Each bar represents the data of the four replicates mean ± SEM of three independent experiments. Significance was shown as ^*^^*^^*^P < 0.001 compared to control, ^###^*P* < 0.001 compared to NAC alone, ^•^*P* < 0.05, ^••^*P* < 0.01, ^•••^*P* < 0.001 compared to As alone groups. As; sodium arsenite, NAC; N-acetylcysteine.

Cellular antioxidant enzyme activities measured after 24 h of exposure to As and NAC were shown in Fig. 3. CAT, SOD, GST, and GPx activities in embryonic fibroblast cells decreased significantly when As and control groups were compared (excluding the 0.01 μM concentration for CAT) (^*^^*^^*^P < 0.001). NAC at a concentration of 10 μM was also found to significantly increase the activities of all enzymes (^•^*P* < 0.05).

### NAC inhibited cell apoptosis induced by As

**Fig. 4:**
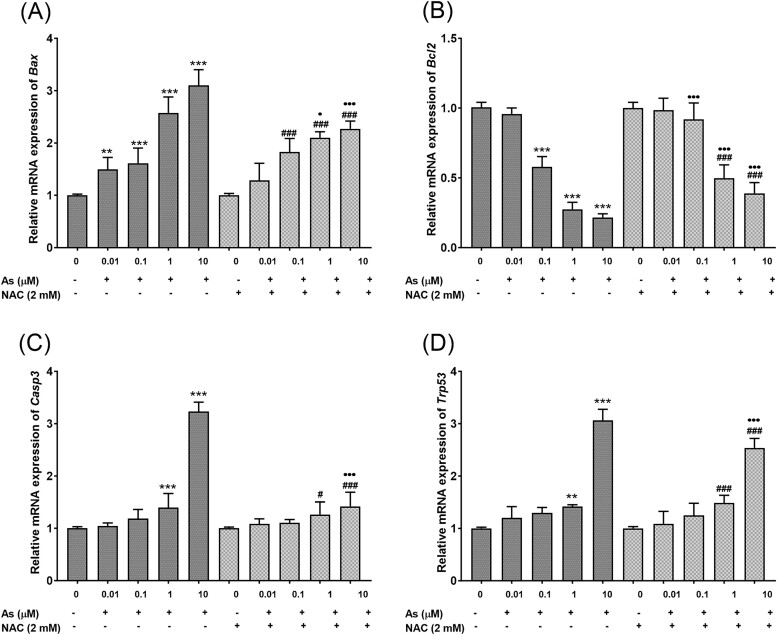
Effects of As and NAC on the mRNA expression of *Bax* (A), *Bcl2* (B), *Casp3* (C), and *Trp53* (D) in embryonic fibroblast cells. Each data point represents the mean ± SEM of three independent experiments carried out in triplicate. Significance was shown as ^*^^*^P < 0.01, ^*^^*^^*^P < 0.001 compared to the control, ^#^*P* < 0.05, ^###^*P* < 0.001 compared to NAC alone, ^•^*P* < 0.05, ^•••^*P* < 0.001 compared to As alone groups. As; sodium arsenite, NAC; N-acetylcysteine.

After the exposure of embryonic fibroblast cells to As and NAC, the expression of apoptotic genes *Bax, Bcl2, Casp3* and *Trp53* was measured by RT-PCR using SYBR Green I fluorescence staining. All the results obtained were normalized according to the housekeeping gene β*-actin*. Gene expression of *Bax* increased significantly at all concentrations in the As exposure groups (Fig. 4A) (*P* < 0.01 and *P* < 0.001), while following co-treatment with As and NAC at 1 and 10 μM concentrations, NAC suppressed the expression of the *Bax* gene (*P* < 0.05 and *P* < 0.001). In the As exposed groups, the expression of *Bcl2* gene was observed to decrease significantly at concentrations of 0.1, 1, and 10 μM concentrations (Fig. 4B) (*P* < 0.001); whereas co-treatment with NAC increased the expression of the *Bcl2* gene at concentrations of 0.1, 1 and 10 μM when NAC and As (*P* < 0.001). *Casp3* gene expression following As exposure increased significantly at 1 and 10 μM concentrations (Fig. 4C) (*P* < 0.001). In the combined exposure of NAC and As, NAC suppressed *Casp3* expression only at the 10 μM concentration (*P* < 0.001). The expression of the *Trp53* gene increased significantly at concentrations of 1 and 10 μM As (Fig. 4D) (*P* < 0.05). In the groups in which As and NAC were administered together, the suppressive effect of NAC on *Casp3* expression was found to be significant only at a concentration of 10 μM (*P* < 0.05).

**Fig. 5:**
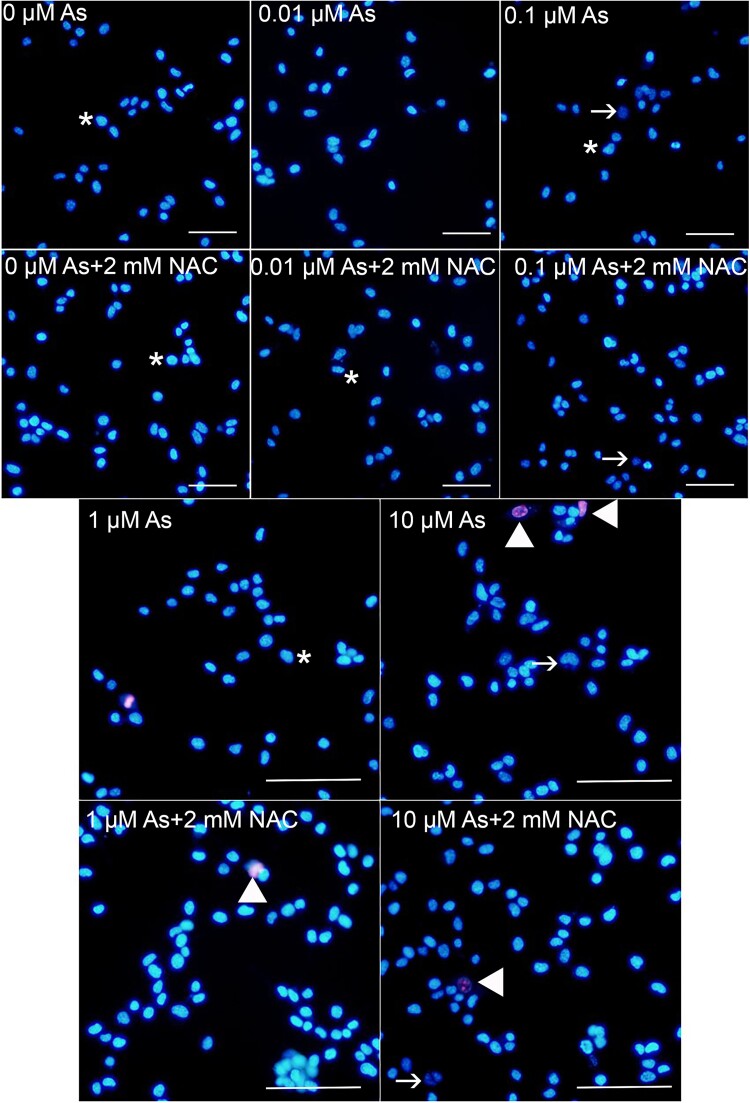
The morphological effect of As and NAC on apoptosis after 24 h exposure in 3T3 cells. →: Apoptotic cells, ►: Dead cells, *: Viable cells.

Viable, apoptotic, and dead 3T3 cells treated with As and/or NAC were evaluated by double fluorescent staining (Fig. 5). The viability of stained cells was determined based on their level of blue fluorescence. Cells with high blue fluorescence were considered viable, while cells with low fluorescence were identified as apoptotic. Additionally, cells with high red fluorescence were classified as dead. As shown in Table 2, when cells exposed to As were analyzed, it was observed that the number of viable cells decreased significantly and the number of apoptotic cells and dead cells increased at concentrations of 0.1, 1, and 10 μM concentrations (*P* < 0.05). Cells treated with NAC showed an improvement in the number of viable cells and apoptotic cells following treatment with 1 and 10 μM concentrations of As + NAC, while the improvement in the number of dead cells was detected only at the concentration of 10 μM (*P* < 0.05).

**Table 2:** Effects of As and NAC on viable, apoptotic, and dead cells depending on concentrations.

**Groups**	**Concentrations (μM)**	**Viable cells**	**Apoptotic cells**	**Dead cells**
As	0 0.01 0.1 1 10	98.40 ± 0.70 97.50 ± 2.09 95.00 ± 1.76^*^ 86.60 ± 3.06^*^ 85.30 ± 1.12^*^	0.38 ± 0.11 0.90 ± 0.34 2.63 ± 0.30^*^ 7.80 ± 0,79^*^ 8.98 ± 0,38^*^	0.55 ± 0.07 0.65 ± 0.12 1.04 ± 0.03^*^ 1.14 ± 0.07^*^ 1.49 ± 0.10^*^
As+NAC	0 0.01 0.1 1 10	98.50 ± 1.09 98.20 ± 0.74 96.70 ± 0.97 95.60 ± 1.13^#,•^ 91.10 ± 0.74^#^	0.37 ± 0.10 0.67 ± 0.16 1.86 ± 0.25 3.00 ± 0.36^#,•^ 6.14 ± 0.70^#^	0.41 ± 0,08 0.50 ± 0,07 0.96 ± 0,05^#^ 1.00 ± 0,04^#,•^ 1.10 ± 0,05^#^

Each data point represents the mean ± standard error of the mean of three independent experiments carried out in triplicate. Significance was shown as *P < 0.05 compared to the control, ^#^*P* < 0.05 compared to NAC alone, ^•^*P* < 0.05, compared to As alone groups. As; sodium arsenite, NAC; N-acetylcysteine.

## Discussion

Arsenic is a toxic substance that has been used in medicine, industry, agriculture, and many other fields for many years. It is naturally found in the Earth’s crust, and its exposure cannot be avoided.^2^^,^^4^ Although arsenic has been investigated for many years, its toxicity has not been fully elucidated. NAC is an important antioxidant for cells in terms of direct scavenging of free radicals and supporting glutathione and cysteine activities.^34^ In this study, we investigated the protective effects of NAC against As cytotoxicity, oxidative stress, and apoptosis in embryonic fibroblast cells. Yedjou et al.^34^ have investigated the cell viability effects of arsenic trioxide and NAC on human leukemia (HL-60) cells for 24 h. The cell viability decreased using 6 μg/mL of arsenic trioxide, while using 25–100 μM NAC along with arsenic trioxide increased the cell viability. He et al.^35^ conducted with Oline-neu cells, it was observed that cell viability decreased as a result of the application of 8–20 μM sodium arsenic, while the viability increased in the application of 8 μM sodium arsenic and 0.5–2 mM NAC. Similar to the studies mentioned above, our research found that the administration of 1 and 10 μM of As dramatically decreased cell viability. However, the treatment of 2 mM NAC prevented the harmful effects of arsenic on cell viability.

The LDH enzyme in the cytoplasm is secreted into the cell culture supernatant following damage to the plasma membrane caused by apoptosis, necrosis, and other cellular damage.^36^ Zhong et al.^37^ conducted a study with arsenic trioxide on hepatocyte (NCTC1469) cells and determined that 18 μM of arsenic trioxide increased the amount of LDH, whereas the same concentration of arsenic trioxide and 1 mM of NAC administration were found to be ameliorative. Our investigation revealed that LDH levels exhibited a rise at As concentrations of 0.1, 1, and 10 μM. Furthermore, the administration of a combination of 10 μM As and 2 mM NAC resulted in a reduction of LDH levels. The data obtained in our study, consistent with the current literature, reveal that NAC may be an important antioxidant against the cytotoxic effect of As.

ROS formed by arsenic exposure cause oxidative damage to proteins, DNA, and similar cell components.^38^ The amount of ROS measured as a result of studies in mouse Oli-neu cells (8 μM As) and embryonic fibroblast cells (0.01–10 μM As) in the literature is similar to the results of our study.^35^^,^^39^ In this study, we found that OH• formation and lipid peroxidation increased with As exposure. When As (10 μM) and/or NAC (2 mM) were applied to cells, NAC reduced both OH• formation and lipid peroxidation. Overall, the results of our investigation correlate with previous findings in the literature, which demonstrated that NAC effectively reduces oxidative stress in mouse Oli-neu cells and human HL-60 cells.^34^^,^^35^

Antioxidant enzymes eliminate ROS-induced cell damage.^40^^,^^41^ This study showed that, upon exposure to arsenic, antioxidant enzymes were insufficient to eliminate ROS in embryonic fibroblast cells. In our study, the activity of CAT enzyme decreased significantly in the presence of As concentrations ranging from 0.1 μM to 10 μM. This result was supported by previous studies with arsenic in osteosarcoma cells and human keratinocyte cell lines.^42^^,^^43^ The combined treatment of As (10 μM) and NAC produced an ameliorative effect. Our results were consistent with previous research that has examined various antioxidants.^39^ In this study, the SOD enzyme activities decreased significantly following exposure to 0.01–10 μM As. These findings are in parallel with previous studies with human liver and human fibroblast cells.^44^ Our investigation supports the positive impact of NAC on SOD activity, which mitigates the inhibitory effects of As. This discovery correlates with the results reported by Messarah et al.^45^ in their research on mouse oligodendrocyte precursor cells. According to the GST enzyme data obtained from our study, it was revealed that As significantly reduced the GST enzyme activity, and NAC inhibited these negative effects of As. Examining the literature revealed that arsenic toxicity in fibroblast and breast tumor cells led to a decrease in GST enzyme activity.^46^ Previous studies found that different antioxidants, such as curcumin, increased the activity of GST following arsenic exposure.^47^ Our current study significantly decreased the GPx enzyme at all As concentrations. Furthermore, treatment with NAC significantly increased the activity of GPx at a concentration of 10 μM As. Previous studies provide evidence that arsenic reduces the activity of GPx, and exposure to NAC application increases the activity of GPx in the hepatoma cell line.^48^ This study, in contrast to previous studies, demonstrated that even at extremely low concentrations, such as 0.01 μM concentration, As has a detrimental impact on the antioxidant enzyme system and disrupts the equilibrium between oxidants and antioxidants. Furthermore, it was discovered that NAC exhibited therapeutic capabilities in the context of arsenic toxicity.

It is well known that increased oxidative stress damages various structures within the cell and causes changes in the DNA sequence.^49^ Many studies have shown that apoptosis-induced death occurs in response to DNA damage.^50^ The irreparable DNA damage created by ROS leads to the activation of *Trp53*, which is also called the genome protector. In this case, ROS causes increased expression of the *Bax* gene, which targets mitochondria and induces apoptosis.^51^*Bcl2* promotes mitochondrial integrity with its anti-apoptotic effects and protects cells from apoptosis.^52^^,^^53^ Caspase-3 induces apoptosis and triggers the release of cytochrome C, leading to the formation of apoptosomes.^54^ In this study, it was determined that the expression of the *Bcl2* gene decreased and the *Bax*, *Casp3, and Trp53* genes increased on exposure to As. As a result of evaluating the literature, the Trp53 protein and caspase-3 activity increased when 50 μM arsenic trioxide was applied to human pulmonary fibroblast cells. In addition, it was observed that *Bax*, *Casp3*, and *Trp53* expressions increased by applying 2–5 μM of arsenic trioxide to pancreatic β-cells, and the apoptotic index increased by applying 1–3 μM of arsenic trioxide to the human glioblastoma cancer cell line.^13^^,^^55^^,^^56^ In a study conducted with Neuro-2a cells, the healing effect of NAC was determined as a result of the co-administration of 5 mM NAC and 5 and 10 μM arsenic trioxide.^57^ In our study, it was observed that the number of apoptotic cells decreased when As and NAC were applied together to embryonic fibroblast cells. Moreover, NAC was found to down-regulate the expression of genes that induce apoptosis (*Bax*, *Trp53,* and *Casp3*) and up-regulate the expression of the anti-apoptotic gene *Bcl2* in embryonic fibroblast cells.

## Conclusions

Consequently, As induces cytotoxicity, suppresses the antioxidant enzyme system, and increases the generation of ROS in embryonic fibroblast cells. As a result of these damages caused by arsenic exposure, the apoptotic process is triggered in the cell. NAC can significantly reduce the cytotoxic, oxidative, and apoptotic effects of As. Furthermore, despite the limitations on the use of arsenic and its compounds in many production processes and the WHO's set limit of 10 μg/L for arsenic in drinking water, it is clear that even at low concentrations employed in our study, it has detrimental effects at the cellular level. NAC is one of the options we must use to mitigate the negative consequences of arsenic exposure for people in many parts of the world.

## References

[1] Tchounwou PB , Wilson B, Ishaque A. Important considerations in the development of public health advisories for arsenic and arsenic-containing compounds in drinking water. Rev Environ Health. 1999:14(4):211–229. 10746734 10.1515/reveh.1999.14.4.211

[2] Muthumani M , Miltonprabu S. Ameliorative efficacy of tetrahydrocurcumin against arsenic induced oxidative damage, dyslipidemia and hepatic mitochondrial toxicity in rats. Chem Biol Interact. 2015:235:95–105. 25869292 10.1016/j.cbi.2015.04.006

[3] Asere TG , Stevens CV, Du Laing G. Use of (modified) natural adsorbents for arsenic remediation: a review. Sci Total Environ. 2019:676:706–720. 31054415 10.1016/j.scitotenv.2019.04.237

[4] Ince S , Avdatek F, Demirel HH, Arslan-Acaroz D, Goksel E, Kucukkurt I. Ameliorative effect of polydatin on oxidative stress-mediated testicular damage by chronic arsenic exposure in rats. Andrologia. 2016:48(5):518–524. 26302725 10.1111/and.12472

[5] Shieh P , Jan CR, Liang WZ. The protective effects of the antioxidant N-acetylcysteine (NAC) against oxidative stress-associated apoptosis evoked by the organophosphorus insecticide malathion in normal human astrocytes. Toxicology. 2019:417:1–14. 30769050 10.1016/j.tox.2019.02.004

[6] Jomova K , Valko M. Advances in metal-induced oxidative stress and human disease. Toxicology. 2011:283(2–3):65–87. 21414382 10.1016/j.tox.2011.03.001

[7] Flora SJ , Bhadauria S, Kannan GM, Singh N. Arsenic induced oxidative stress and the role of antioxidant supplementation during chelation: a review. J Environ Biol. 2007:28(2 Suppl):333–347. 17929749

[8] Luo F , Zhuang Y, Sides MD, Sanchez CG, Shan B, White ES, Lasky JA. Arsenic trioxide inhibits transforming growth factor-beta1-induced fibroblast to myofibroblast differentiation in vitro and bleomycin induced lung fibrosis in vivo. Respir Res. 2014:15(1):51. 24762191 10.1186/1465-9921-15-51 PMC4113202

[9] Zhong L , Hao H, Chen D, Hou Q, Zhu Z, He W, Sun S, Sun M, Li M, Fu X. Arsenic trioxide inhibits the differentiation of fibroblasts to myofibroblasts through nuclear factor erythroid 2-like 2 (NFE2L2) protein and the Smad2/3 pathway. J Cell Physiol. 2019:234(3):2606–2617. 30317545 10.1002/jcp.27073

[10] Oya-Ohta Y , Kaise T, Ochi T. Induction of chromosomal aberrations in cultured human fibroblasts by inorganic and organic arsenic compounds and the different roles of glutathione in such induction. Mutat Res. 1996:357(1–2):123–129. 8876688 10.1016/0027-5107(96)00092-9

[11] You BR , Park WH. Arsenic trioxide induces human pulmonary fibroblast cell death via increasing ROS levels and GSH depletion. Oncol Rep. 2012:28(2):749–757. 22684917 10.3892/or.2012.1852

[12] Li C , Qu X, Xu W, Qu N, Mei L, Liu Y, Wang X, Yu X, Liu Z, Nie D, et al. Arsenic trioxide induces cardiac fibroblast apoptosis in vitro and in vivo by up-regulating TGF-beta1 expression. Toxicol Lett. 2013:219(3):223–230. 23542815 10.1016/j.toxlet.2013.03.024

[13] Park WH , Kim SH. Arsenic trioxide induces human pulmonary fibroblast cell death via the regulation of Bcl-2 family and caspase-8. Mol Biol Rep. 2012:39(4):4311–4318. 21779797 10.1007/s11033-011-1218-z

[14] Tardiolo G , Bramanti P, Mazzon E. Overview on the effects of N-Acetylcysteine in neurodegenerative diseases. Molecules. 2018:23(12):3305. 10.3390/molecules23123305 PMC6320789 30551603

[15] Dhouib IE , Jallouli M, Annabi A, Gharbi N, Elfazaa S, Lasram MM. A minireview on N-acetylcysteine: an old drug with new approaches. Life Sci. 2016:151:359–363. 26946308 10.1016/j.lfs.2016.03.003

[16] Li GX , Chen YK, Hou Z, Xiao H, Jin H, Lu G, Lee MJ, Liu B, Guan F, Yang Z, et al. Pro-oxidative activities and dose-response relationship of (−)-epigallocatechin-3-gallate in the inhibition of lung cancer cell growth: a comparative study in vivo and in vitro. Carcinogenesis. 2010:31(5):902–910. 20159951 10.1093/carcin/bgq039 PMC2864413

[17] Kerksick C , Willoughby D. The antioxidant role of glutathione and N-acetyl-cysteine supplements and exercise-induced oxidative stress. J Int Soc Sports Nutr. 2005:2(2):1–7. 18500954 10.1186/1550-2783-2-2-38 PMC2129149

[18] Kelly GS . Clinical applications of N-acetylcysteine. Altern Med Rev. 1998:3(2):114–127. 9577247

[19] Zafarullah M , Li WQ, Sylvester J, Ahmad M. Molecular mechanisms of N-acetylcysteine actions. Cell Mol Life Sci. 2003:60(1):6–20. 12613655 10.1007/s000180300001 PMC11138873

[20] Millea PJ . N-acetylcysteine: multiple clinical applications. Am Fam Physician. 2009:80(3):265–269. 19621836

[21] Gartner LP , Hiatt JL. Color atlas and text of histology. Baltimore, MD: Lippincott Williams Wilkins; 2012.

[22] Young B , Woodford P, O'Dowd G. Wheater's functional histology E-book: a text and colour atlas. Philadelphia, PA: Elsevier Health Sci; 2013.

[23] Gomez-Caminero A , Howe P, Hughes M, Kenyon E, Lewis DR, Moore M, Ng J, Aitio A, Becking G. Environmental health criteria 224 arsenic and arsenic compounds. 2nd ed. Geneva: World Health Organization; 2001.

[24] Petrusevski B , Sharma S, Schippers JC. Arsenic in drinking water. Vol. 17. Oxford, UK: Delft: IRC International Water and Sanitation Center; 2007 pp. 36–44.

[25] Peng YW , Buller CL, Charpie JR. Impact of N-acetylcysteine on neonatal cardiomyocyte ischemia-reperfusion injury. Pediatr Res. 2011:70(1):61–66. 21427628 10.1203/PDR.0b013e31821b1a92

[26] Wang L , Xu Y, Zhao X, Zhu X, He X, Sun A, Zhuang G. Antagonistic effects of N-acetylcysteine on lead-induced apoptosis and oxidative stress in chicken embryo fibroblast cells. Heliyon. 2023:9(11):e21847. 38034812 10.1016/j.heliyon.2023.e21847 PMC10682149

[27] Puntarulo S , Cederbaum AI. Comparison of the ability of ferric complexes to catalyze microsomal chemiluminescence, lipid peroxidation, and hydroxyl radical generation. Arch Biochem Biophys. 1988:264(2):482–491. 2840858 10.1016/0003-9861(88)90313-x

[28] Devasagayam TP , Tarachand U. Pregnancy-associated decrease in lipid peroxidation in rat liver. Biochem Int. 1988:16(1):45–52. 3355575

[29] Marklund S , Marklund G. Involvement of the superoxide anion radical in the autoxidation of pyrogallol and a convenient assay for superoxide dismutase. Eur J Biochem. 1974:47(3):469–474. 4215654 10.1111/j.1432-1033.1974.tb03714.x

[30] Sinha AK . Colorimetric assay of catalase. Anal Biochem. 1972:47(2):389–394. 4556490 10.1016/0003-2697(72)90132-7

[31] Hafeman DG , Sunde RA, Hoekstra WG. Effect of dietary selenium on erythrocyte and liver glutathione peroxidase in the rat. J Nutr. 1974:104(5):580–587. 4823943 10.1093/jn/104.5.580

[32] Habig WH , Pabst MJ, Fleischner G, Gatmaitan Z, Arias IM, Jakoby WB. The identity of glutathione S-transferase B with ligandin, a major binding protein of liver. Proc Natl Acad Sci USA. 1974:71(10):3879–3882. 4139704 10.1073/pnas.71.10.3879 PMC434288

[33] Livak KJ , Schmittgen TD. Analysis of relative gene expression data using real-time quantitative PCR and the 2− ΔΔCT method. Methods. 2001:25(4):402–408. 11846609 10.1006/meth.2001.1262

[34] Yedjou CG , Rogers C, Brown E, Tchounwou PB. Differential effect of ascorbic acid and n-acetyl-L-cysteine on arsenic trioxide-mediated oxidative stress in human leukemia (HL-60) cells. J Biochem Mol Toxicol. 2008:22(2):85–92. 18418892 10.1002/jbt.20223 PMC2678234

[35] He Z , Zhang Y, Zhang H, Zhou C, Ma Q, Deng P, Lu M, Mou Z, Lin M, Yang L, et al. NAC antagonizes arsenic-induced neurotoxicity through TMEM179 by inhibiting oxidative stress in Oli-neu cells. Ecotoxicol Environ Saf. 2021:223:112554. 34332247 10.1016/j.ecoenv.2021.112554

[36] Bruni A , Pepper AR, Pawlick RL, Gala-Lopez B, Gamble AF, Kin T, Seeberger K, Korbutt GS, Bornstein SR, Linkermann A, et al. Ferroptosis-inducing agents compromise in vitro human islet viability and function. Cell Death Dis. 2018:9(6):595. 29789532 10.1038/s41419-018-0506-0 PMC5964226

[37] Zhong G , Wan F, Ning Z, Wu S, Jiang X, Tang Z, Huang R, Hu L. The protective role of autophagy against arsenic trioxide-induced cytotoxicity and ROS-dependent pyroptosis in NCTC-1469 cells. J Inorg Biochem. 2021:217:111396. 33610032 10.1016/j.jinorgbio.2021.111396

[38] Ding W , Hudson LG, Liu KJ. Inorganic arsenic compounds cause oxidative damage to DNA and protein by inducing ROS and RNS generation in human keratinocytes. Mol Cell Biochem. 2005:279(1–2):105–112. 16283519 10.1007/s11010-005-8227-y

[39] Perker MC , Orta Yilmaz B, Yildizbayrak N, Aydin Y, Erkan M. Protective effects of curcumin on biochemical and molecular changes in sodium arsenite-induced oxidative damage in embryonic fibroblast cells. J Biochem Mol Toxicol. 2019:33(7):e22320. 30934151 10.1002/jbt.22320

[40] Kabir MT , Rahman MH, Shah M, Jamiruddin MR, Basak D, al-Harrasi A, Bhatia S, Ashraf GM, Najda A, el-kott AF, et al. Therapeutic promise of carotenoids as antioxidants and anti-inflammatory agents in neurodegenerative disorders. Biomed Pharmacother. 2022:146:112610. 35062074 10.1016/j.biopha.2021.112610

[41] Mohammed ET , Hashem KS, Ahmed AE, Aly MT, Aleya L, Abdel-Daim MM. Ginger extract ameliorates bisphenol a (BPA)-induced disruption in thyroid hormones synthesis and metabolism: involvement of Nrf-2/HO-1 pathway. Sci Total Environ. 2020:703:134664. 31757552 10.1016/j.scitotenv.2019.134664

[42] Sun X , Li B, Li X, Wang Y, Xu Y, Jin Y, Piao F, Sun G. Effects of sodium arsenite on catalase activity, gene and protein expression in HaCaT cells. Toxicol in Vitro. 2006:20(7):1139–1144. 16600567 10.1016/j.tiv.2006.02.008

[43] Wang Y , Wei Y, Zhang H, Shi Y, Li Y, Li R. Arsenic trioxide induces apoptosis of p53 null osteosarcoma MG63 cells through the inhibition of catalase. Med Oncol. 2012:29(2):1328–1334. 21308489 10.1007/s12032-011-9848-5

[44] Ahamed M , Akhtar MJ, Alhadlaq HA. Co-exposure to SiO2 nanoparticles and arsenic induced augmentation of oxidative stress and mitochondria-dependent apoptosis in human cells. Int J Environ Res Public Health. 2019:16(17):3199. 10.3390/ijerph16173199 PMC6747183 31480624

[45] Messarah M , Klibet F, Boumendjel A, Abdennour C, Bouzerna N, Boulakoud MS, el Feki A. Hepatoprotective role and antioxidant capacity of selenium on arsenic-induced liver injury in rats. Exp Toxicol Pathol. 2012:64(3):167–174. 20851583 10.1016/j.etp.2010.08.002

[46] Schuliga M , Chouchane S, Snow ET. Upregulation of glutathione-related genes and enzyme activities in cultured human cells by sublethal concentrations of inorganic arsenic. Toxicol Sci. 2002:70(2):183–192. 12441363 10.1093/toxsci/70.2.183

[47] El-Demerdash FM , Yousef MI, Radwan FM. Ameliorating effect of curcumin on sodium arsenite-induced oxidative damage and lipid peroxidation in different rat organs. Food Chem Toxicol. 2009:47(1):249–254. 19049818 10.1016/j.fct.2008.11.013

[48] Selvaraj V , Yeager-Armstead M, Murray E. Protective and antioxidant role of selenium on arsenic trioxide-induced oxidative stress and genotoxicity in the fish hepatoma cell line PLHC-1. Environ Toxicol Chem. 2012:31(12):2861–2869. 23023949 10.1002/etc.2022

[49] Sharma V , Singh P, Pandey AK, Dhawan A. Induction of oxidative stress, DNA damage and apoptosis in mouse liver after sub-acute oral exposure to zinc oxide nanoparticles. Mutat Res. 2012:745(1–2):84–91. 22198329 10.1016/j.mrgentox.2011.12.009

[50] Mates JM , Segura JA, Alonso FJ, Márquez J. Oxidative stress in apoptosis and cancer: an update. Arch Toxicol. 2012:86(11):1649–1665. 22811024 10.1007/s00204-012-0906-3

[51] Hori YS , Kuno A, Hosoda R, Horio Y. Regulation of FOXOs and p53 by SIRT1 modulators under oxidative stress. PLoS One. 2013:8(9):e73875. 24040102 10.1371/journal.pone.0073875 PMC3770600

[52] Maes ME , Schlamp CL, Nickells RW. BAX to basics: how the BCL2 gene family controls the death of retinal ganglion cells. Prog Retin Eye Res. 2017:57:1–25. 28064040 10.1016/j.preteyeres.2017.01.002 PMC5350025

[53] Edlich F . BCL-2 proteins and apoptosis: recent insights and unknowns. Biochem Biophys Res Commun. 2018:500(1):26–34. 28676391 10.1016/j.bbrc.2017.06.190

[54] Grilo AL , Mantalaris A. Apoptosis: a mammalian cell bioprocessing perspective. Biotechnol Adv. 2019:37(3):459–475. 30797096 10.1016/j.biotechadv.2019.02.012

[55] Lu TH , Su CC, Chen YW, Yang CY, Wu CC, Hung DZ, Chen CH, Cheng PW, Liu SH, Huang CF. Arsenic induces pancreatic beta-cell apoptosis via the oxidative stress-regulated mitochondria-dependent and endoplasmic reticulum stress-triggered signaling pathways. Toxicol Lett. 2011:201(1):15–26. 21145380 10.1016/j.toxlet.2010.11.019

[56] Dizaji MZ , Malehmir M, Ghavamzadeh A, Alimoghaddam K, Ghaffari SH. Synergistic effects of arsenic trioxide and silibinin on apoptosis and invasion in human glioblastoma U87MG cell line. Neurochem Res. 2012:37(2):370–380. 21969006 10.1007/s11064-011-0620-1

[57] Lu TH , Tseng TJ, Su CC, Tang FC, Yen CC, Liu YY, Yang CY, Wu CC, Chen KL, Hung DZ, et al. Arsenic induces reactive oxygen species-caused neuronal cell apoptosis through JNK/ERK-mediated mitochondria-dependent and GRP 78/CHOP-regulated pathways. Toxicol Lett. 2014:224(1):130–140. 24157283 10.1016/j.toxlet.2013.10.013

